# Microstructure-based constitutive model of coronary artery with active smooth muscle contraction

**DOI:** 10.1038/s41598-017-08748-7

**Published:** 2017-08-24

**Authors:** H. Chen, G. S. Kassab

**Affiliations:** California Medical Innovations Institute, Inc., San Diego, CA92121 USA

## Abstract

Currently, there is no full three-dimensional (3D) microstructural mechanical model of coronary artery based on measured microstructure including elastin, collagen and smooth muscle cells. Many structural models employ mean values of vessel microstructure, rather than continuous distributions of microstructure, to predict the mechanical properties of blood vessels. Although some models show good agreements on macroscopic vessel responses, they result in a lower elastin stiffness and earlier collagen recruitment. Hence, a full microstructural constitutive model is required for better understanding vascular biomechanics in health and disease. Here, a 3D microstructural model that accounts for all constituent microstructure is proposed to predict macroscopic and microscopic responses of coronary arteries. Coronary artery microstructural parameters were determined based on previous statistical measurements while mechanical testing of arteries (n = 5) were performed in this study to validate the computational predictions. The proposed model not only provides predictions of active and passive stress distributions of vessel wall, but also enables reliable estimations of material parameters of individual fibers and cells and thus predicts microstructural stresses. The validated microstructural model of coronary artery sheds light on vascular biomechanics and can be extend to diseased vessels for better understanding of initiation, progression and clinical treatment of vascular disease.

## Introduction

An in-depth understanding of mechanical properties of coronary arteries is essential for elucidating the mechanism of initiation and progression of vascular disease^1–3^. For a healthy vessel, the principle function of smooth muscle cells (SMCs) is to maintain vascular tone and resistance (in the case of small arteries). The reinforcement of SMCs by the extracellular matrix (i.e., elastin and collagen fibers) ensures that the cells can withstand the imposed loads due to hemodynamic forces. Perturbation of stress or stretch on vessel wall (e.g., hypo- or hyper-tension, flow increase or decrease, etc.), however, activates various heparanases and a cascade of proteases that influence the adhesion of extracellular matrix to SMC surface, providing the trigger for cell phenotypic changes along with vascular growth and remodeling^2, 4^. Thus, prediction of stresses on individual cells and fibers is significant but has been a major challenge for mechanical modeling of blood vessels. Microstructure-based constitutive models have been advanced to predict both macro-and micro-level mechanical environments in blood vessel wall in recent years.

Microstructural models can provide accurate predictions if based on realistic microstructural data. The majority of microstructural models have focused on the passive properties of blood vessels that are mainly determined by elastin and collagen fibers^5–8^. Many studies consider the vessel wall as a composite of elastin and collagen fibers embedded in a fluid-like matrix^1, 3, 9–11^. The fibers are the only constituent phases that sustain non-hydrostatic loading, such as tension and shear, whereas the contribution of the fluid-like matrix is only a hydrostatic pressure. The fluid-like matrix approach allows affine deformation of the microstructure that may involve any geometrical distributions of fibers, such as orientation and undulation distributions. Based on microstructural features in coronary media, Hollander *et al*. showed that an affine model provided good predictions of coronary media twist response based on parameters estimated from biaxial tests of inflation and extension^3^. In addition, good predictive values were demonstrated for the model behavior at high axial stretch ratio based on data of low stretches. Recently, Chen *et al*. developed a 3D microstructural model for coronary adventitia that incorporated realistic elastin and collagen fiber distributions throughout the wall^1^. The model was validated against a set of biaxial (distension-extension) experiments of coronary adventitia and shown to have a high predictive value. The model also enabled a reliable estimation of material parameters of individual fibers that were physically reasonable and in agreement with measurements.

Unlike passive properties of vessel wall, a microstructure-based active mechanical response of blood vessel is limited in the literature. Most studies suggest a blood vessel exhibits a uniaxial vasoconstriction; i.e., contraction is only in the circumferential direction with no axial response, assuming completely circumferentially oriented SMCs^12–15^. Conversely, Lu and Kassab^16^ found considerable axial force changes during carotid and femoral arteries contraction. The study of Hayman *et al*.^17^ also showed that SMC vasoconstriction reduced carotid artery buckling as compared with the passive state, indicating that SMC contraction may shorten the artery in the axial direction. Huo *et al*.^18^ found that axial force significantly increased and outer diameter decreased during K^+^-induced SMC contractions. Hence, they proposed a biaxial active model to incorporate circumferential and axial vasoactivity of coronary arteries. More recently, Zhou *et al*. incorporated a four-fiber passive constitutive model with a biaxial model of SMCs contraction to investigate biaxial active stresses for the porcine primary renal artery^19^, while Takamizawa developed a triaxial constitutive law to describe the multi-axial active mechanical properties of constricted carotid arteries^20^. These models, however, were 2D models as they assumed uniform stress distribution through the wall thickness and they were not microstructure-based models that exclude orientation distributions of SMCs in the media. Chen *et al*. recently measured SMCs orientation of porcine coronary arteries to integrate a biaxial microstructural model of active coronary media, which revealed the biaxial vasoactivity to be induced by oblique SMC arrangement^11^. Although this model accounts for orientation distributions of fibers and cells, it only provided 2D analysis of stress distributions and did not predict stresses on transmural fibers and cells.

The above cited studies of passive and active mechanical properties suggest a need for 3D microstructure model for the entire coronary wall, which accounts for all microstructure constituents including both passive and active responses. A unified fully integrated model is able to predict the arterial biaxial vasoactivity and stress distribution in the vessel wall as well as provide reliable parameter estimations of individual fibers and SMCs. Here, we introduce such a 3D microstructural model of coronary artery that integrates individual fibers and cells, as well as their orientation and undulation distributions in adventitia and media. An asymmetric constitutive model was employed to describe biaxial active properties of a single SMC. Moreover, geometrical distribution parameters were refined by imposing restrictions to ensure fibers/cells follow statistical distributions previously measured, which leads to even better prediction of passive and active responses and thus provides a reliable estimate of material parameters of individual fibers and cells. The estimated material parameters were compared with those of a mean-value approach that eliminates continuous distribution of microstructure to show that the full measured fiber distributions are necessary to obtain realistic material parameters.

## Results

**Figure 1:**
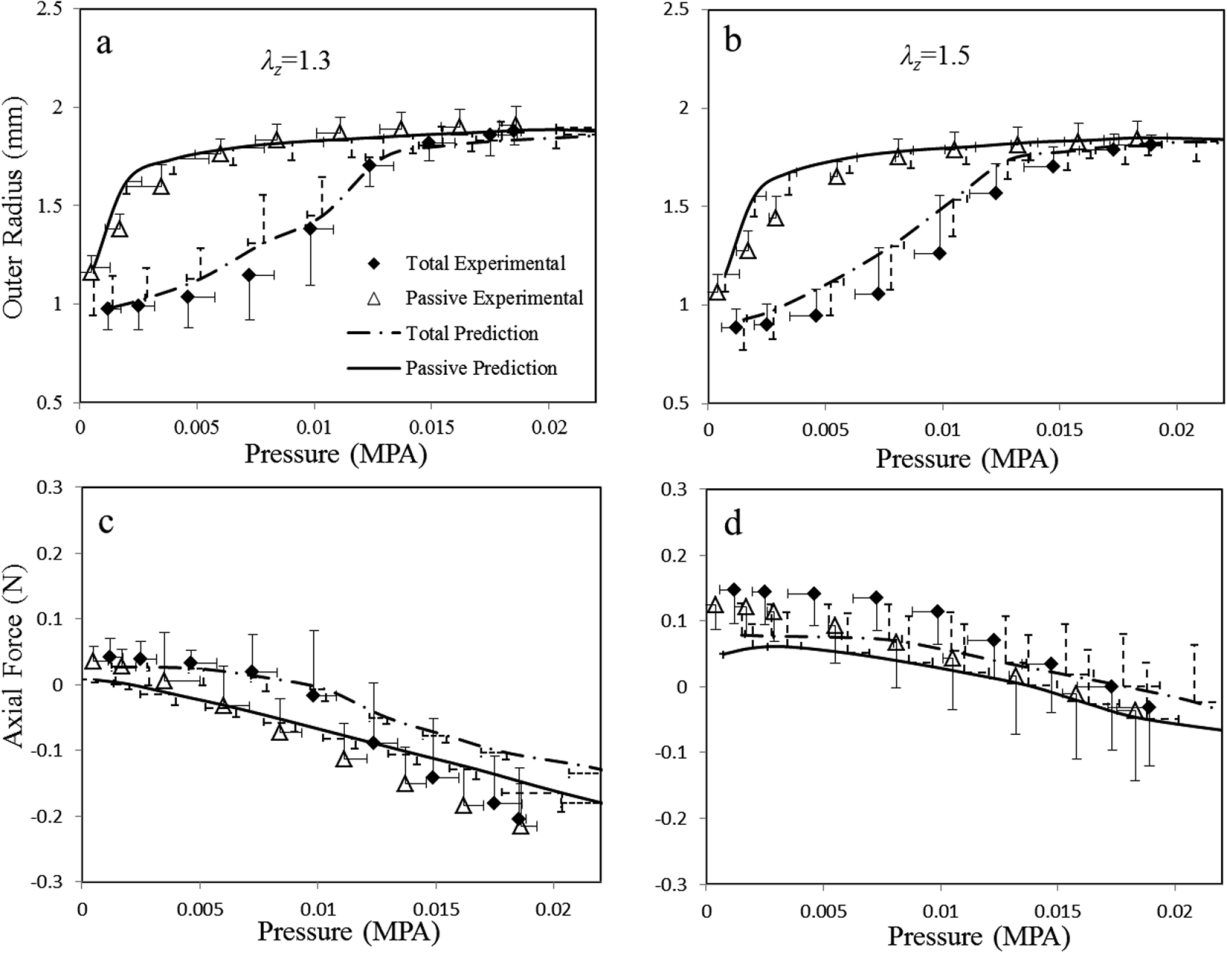
Model predictions with integration of previously measured microstructural distributions. Solid lines denote model predictions of passive responses, and dashed lines denote predictions of full responses of coronary arteries with K^+^ induced SMC contraction. Symbols indicate experiment measurements and error bars denote standard deviation. (**a**) and (**b**) show the pressure-radius relationships at two axial stretch ratios of *λ*_*z*_ = 1.3 and 1.5, respectively, and (**c**) and (**d**) show corresponding pressure-force relationships.

The material parameters of fibers and cells estimated by integration of previously measured microstructural data into the model are provided in Table 1, and the corresponding model predictions of pressure-radius and pressure-force relations are plotted in Fig. 1. The measurements of total (passive and active) and passive arterial responses are compared with model predictions under two different axial stretch ratios *λ*_*z*_ = 1.3 and 1.5, respectively. The predictions are in good agreement with experimental measurements and capture the nonlinear passive nonlinear behavior as well as the biaxial active responses of coronary arteries. The larger axial stretch *λ*_*z*_ = 1.5 leads to smaller diameters and higher axial force. The R^2^ of goodness-of-fit are also provided in Table 1.

**Table 1:** Material parameter estimates of fibers and SMC determined by integration of statistic geometrical distributions of fibers and cells into the model, based on both passive and full distension-extension experimental data of axial stretch ratios *λ*_*z*_ = 1.3 and 1.5.

Parameters		Sample No.					Average ± SD
		1	2	3	4	5	
*Fiber passive*	*k*_*IL*_ (MPa)	0.19	0.05	0.02	0.23	0.17	0.13 ± 0.08
	*k*_*E*_ (MPa)	0.25	0.28	0.13	0.13	0.26	0.21 ± 0.07
	*k*_*C*_ (MPa)	10.0	48.2	10.9	100	36.4	41.1 ± 32.9
	*M*_*C*_	5.96	5.71	6.00	5.99	4.06	5.54 ± 0.75
*Goodness-of-fit of passive data*	*R* ^2^ for *P*	0.80	0.96	0.95	0.96	0.96	0.93 ± 0.06
	*R* ^2^ for *F*	0.78	0.77	0.82	0.72	0.86	0.79 ± 0.05
	*R* ^2^ for *r*_*o*_	0.91	0.91	0.96	0.93	0.94	0.93 ± 0.02
*SMC active*	*ρ*_1_ (MPa)	0.47	0.45	0.63	0.20	0.3	0.41 ± 0.15
	*ρ*_2_	1.22	0.59	0.03	1.66	0.9	0.88 ± 0.55
	*λ*_*max*_	1.44	1.06	1.39	1.38	1.36	1.33 ± 0.14
	${\sigma }_{max}$ (MPa)	0.10	0.11	0.07	0.06	0.12	0.09 ± 0.02
	*τ*	0.14	0.20	0.45	0.87	0.13	0.36 ± 0.28
	*k*_*SMC*_ (MPa)	0.12	0.00	0.003	0.02	0.002	0.03 ± 0.05
*Goodness-of-fit of full data*	*R* ^2^ for *P*	0.83	0.69	0.99	0.99	0.83	0.87 ± 0.11
	*R* ^2^ for *F*	0.71	0.77	0.72	0.78	0.86	0.77 ± 0.05
	*R* ^2^ for *r*_*o*_	0.90	0.58	0.85	0.91	0.91	0.83 ± 0.13

**Figure 2:**
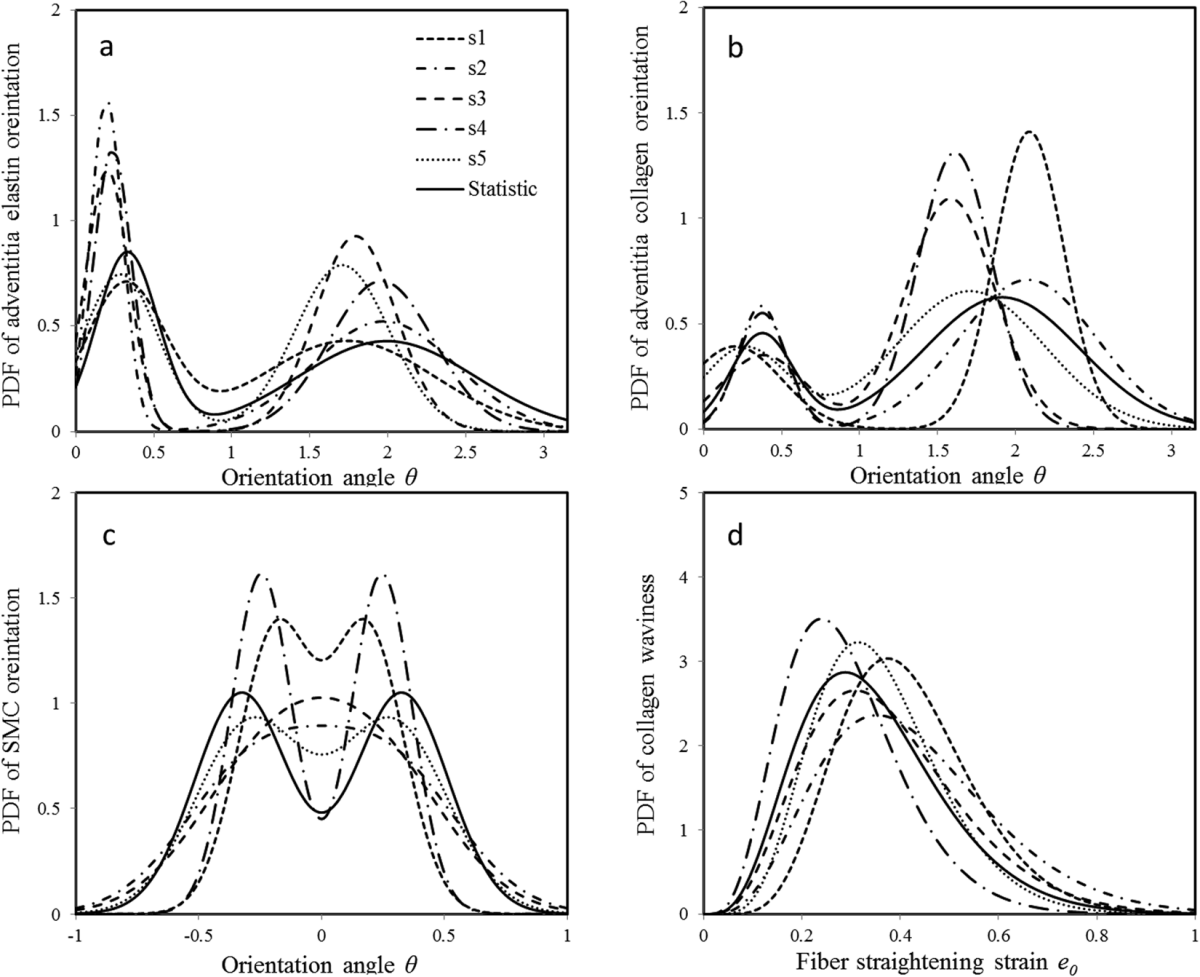
Refined geometric distributions for each sample by parameter optimization. Solid lines denote statistical distribution of previous measurement; dashed lines are distributions for samples #1, #2, #3, #4, #5, respectively. PDF denotes probability density function of a geometrical parameter.

Since there are microstructural variations among samples, geometrical parameters of fibers and cells were refined by imposing restrictions to ensure fiber orientation and waviness follow the measured statistical distributions during process of parameter optimization. The estimated material and geometrical parameters are provided in Table 2. Figure 2 shows refined fibers and cells distributions, of which solid lines denote statistical distributions previously measured on other animal groups, and dashed lines denote refined distributions. Elastin and collagen fibers follow a mixture of two normal distributions showing left/right shifts and tighter disperses compared with those of statistical distributions. For media SMC orientations, samples of #1, #4, and #5 follow two symmetrical distributions against circumferential direction of arteries, while samples of #2 and #3 present approximate single normal distributions as a result of the sum of two symmetrical distributions. Collagen straightening strains follow beta distributions, showing delayed or advanced fiber recruitments as compared with the statistical distribution.

**Table 2:** Material and geometrical parameter estimates of individual elastin, collagen fibers and SMC with refined microstructural geometrical parameters that still follow statistic distributions.

	Parameters	Sample No.					Average ± SD
		1	2	3	4	5	
*Fiber passive*	*k*_*IL*_ (MPa)	0.15	0.04	0.04	0.28	0.39	0.18 ± 0.14
	*k*_*E*_ (MPa)	0.30	0.28	0.14	0.28	0.35	0.27 ± 0.07
	*k*_*C*_ (MPa)	11.8	55.1	10.9	57.9	11.8	29.5 ± 22.1
	*M*_*C*_	5.68	5.65	5.91	5.58	3.32	5.23 ± 0.96
*Fiber geometrical*	*e*_02_	0.39	0.38	0.37	0.38	0.25	0.35 ± 0.05
	*α*_*1*_	8.30	4.97	4.90	5.22	6.97	6.07 ± 1.35
	*α*_*2*_	71.9	42.0	47.7	67.2	71.0	60.0 ± 12.6
	*μ*_*E1*_	0.32	0.20	0.20	0.24	0.29	0.25 ± 0.05
	*σ*_*E1*_	0.26	0.10	0.14	0.12	0.24	0.17 ± 0.07
	*μ*_*E2*_	1.74	1.97	1.80	1.96	1.71	1.84 ± 0.11
	*σ*_*E2*_	0.56	0.46	0.26	0.33	0.30	0.38 ± 0.11
	*μ*_*C1*_	0.20	0.37	0.38	0.38	0.26	0.32 ± 0.07
	*σ*_*C1*_	0.30	0.15	0.27	0.16	0.27	0.23 ± 0.06
	*μ*_*C2*_	2.09	2.08	1.58	1.61	1.71	1.81 ± 0.23
	*σ*_*C2*_	0.22	0.44	0.28	0.24	0.47	0.33 ± 0.10
	*μ*_*M*_	0.29	0.18	0.28	0.13	0.26	0.23 ± 0.06
	*σ*_*M*_	0.30	0.11	0.13	0.16	0.27	0.19 ± 0.08
*SMC active*	$\,{\rho }_{1}$ (MPa)	0.27	0.30	0.47	0.25	0.27	0.31 ± 0.08
	*ρ*_2_	1.04	1.83	1.66	1.83	0.79	1.43 ± 0.43
	*λ*_*max*_	1.45	1.28	1.48	1.35	1.16	1.34 ± 0.12
	σ_*max*_ (MPa)	0.08	0.09	0.08	0.07	0.11	0.09 ± 0.01
	*τ*	0.32	0.11	0.15	0.26	0.30	0.23 ± 0.08
	*k*_*SMC*_ (MPa)	0.03	0.00	0.00	0.03	0.01	0.01 ± 0.01
*SMC geometrical*	*μ*_*SMC*_	0.19	0.27	0.22	0.25	0.29	0.24 ± 0.04
	*σ*_*SMC*_	0.15	0.29	0.29	0.12	0.22	0.21 ± 0.07
*Goodness-of-fit of full data*	*R* ^2^ for *P*	0.95	0.85	0.99	0.99	0.94	0.94 ± 0.05
	*R* ^2^ for *F*	0.83	0.83	0.77	0.93	0.79	0.83 ± 0.06
	*R* ^2^ for *r*_*o*_	0.96	0.93	0.87	0.95	0.98	0.94 ± 0.04

**Figure 3:**
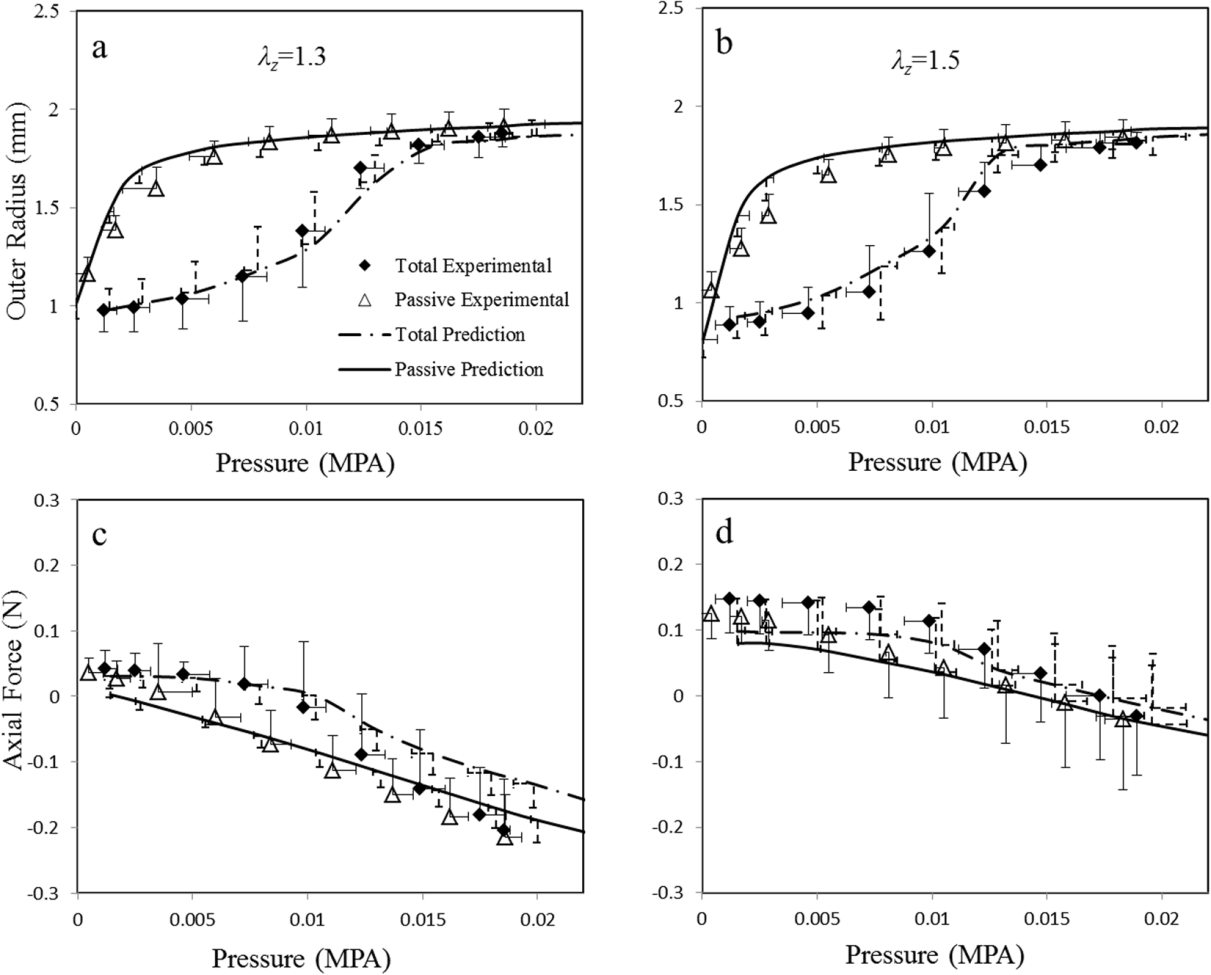
Model predictions with refined microstructural distributions showing better agreements with experimental data. Solid lines denote model predictions of passive responses, and dashed lines denote predictions of full responses of coronary arteries with K^+^ induced SMC contraction. Symbols indicate experiment measurements. (**a**) and (**b**) show the pressure-radius relationships at two axial stretch ratios of *λ*_*z*_ = 1.3 and 1.5, respectively, and (**c**) and (**d**) show corresponding pressure-force relationships.

Figure 3 shows the model incorporating refined microstructure providing better predictions of vessel responses with improved R^2^ (as listed in Table 2). The mean stiffness parameter of IL elastin fibrils was *k*_*IL *_= 0.18 ± 0.14 MPa, similar to that of planer elastin fibers *k*_*E*_ = 0.27 ± 0.07 MPa, while parameters of collagen fiber were *k*_*C*_ = 29.5 ± 22.1 MPa and *M*_*C*_ = 5.23 ± 0.96. These estimated materials parameters of elastin and collagen based on experimental data of the full vessel response, are consistent with those estimated on passive individual adventitia in our recent study; i.e., *k*_*E*_ = 0.19 ± 0.07 MPa, *k*_*C*_ = 27.2 ± 5.1 MPa and *M*_*C*_ = 5.37 ± 0.53^1^. The active material parameters are summarized in Table 2. The optimal stretch ratio for SMCs is *λ*_*max*_ = 1.34 ± 0.12, at which the maximum stress *σ*_*max*_ = 0.09 ± 0.01 MPa is generated. The ratio of axial to circumferential stresses *τ* = *0.23* ± 0.08, indicating that axial active response is significant and cannot be ignored.

**Figure 4:**
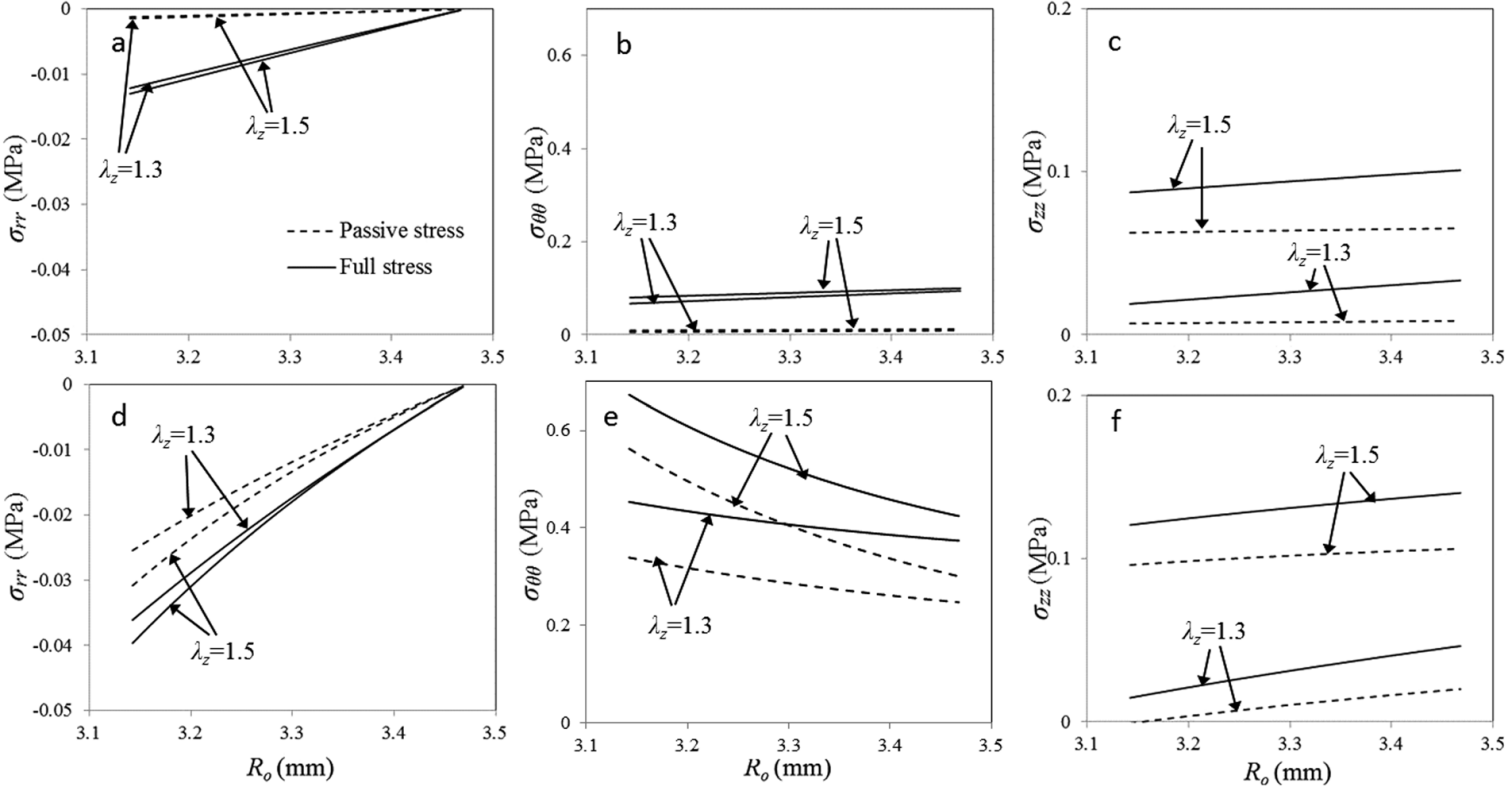
Model predictions of transmural stress distributions at two different circumferential stretch ratios. (**a**–**c**) Full and passive stress components ${\sigma }_{rr}$, ${\sigma }_{rr}$ and ${\sigma }_{zz}$ vary among the vessel wall at a lower circumferential stretch ratio *λ*_*θ*_ = 1.24 with two axial stretch ratios of *λ*_*z*_ = 1.3 and 1.5; (**d**–**f**) the stress components vary among the vessel wall at a higher circumferential ratio ${\lambda }_{\theta }=1.6$.

The transmural stress distributions for the three Cauchy components: radial stress σ_*rr*_, circumferential stress σ_*θθ*_ and axial stress σ_*zz*_, at two different circumferential stretch ratios *λ*_*θ*_ = 1.24 and 1.6 are shown in Fig. 4. The magnitude of radial stress is much smaller than other components. Large strains, either *λ*_*θ*_ or *λ*_*Z*_, lead to greater compression on the luminal side as does SMC contractions. At low circumferential stretch *λ*_*θ*_ = 1.24, passive circumferential stresses σ_*θθ*_ are very low and active SMCs increase total stress. The difference between *λ*_*z*_ = 1.3 and *λ*_*z*_ = 1.5, is negligible. The stresses slightly increase towards the adventitia side. At the high stretch *λ*_*θ*_ = 1.6, both passive and total circumferential stresses increase significantly and larger *λ*_*z*_ prompts higher stresses. Unlike at *λ*_*θ*_ = 1.24, the stresses gradually decrease towards the adventitia side. At the axial direction, σ_*zz*_ are significant even at low circumferential stretch, especially for *λ*_*z*_ = 1.5. The axial stresses slightly increase toward the adventitia side for all cases.

**Figure 5:**
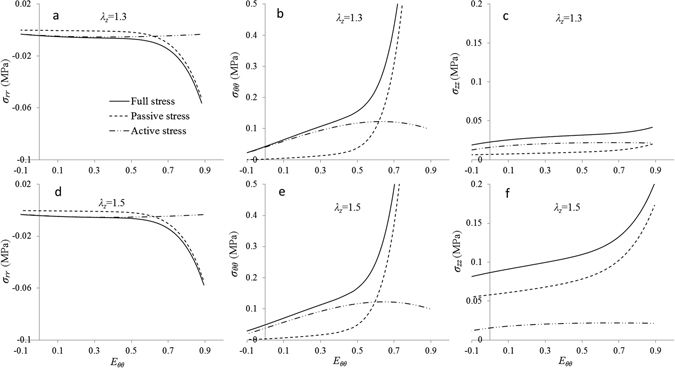
Model predicted stress-strain relation of middle wall of coronary arteries. (**a**–**c**) Full, passive and active stresses of middle wall vary with increase of circumferential strain at a fixed axial stretch ratio of *λ*_*z*_ = 1.3; (**d–f**). Full, passive and active stresses of middle wall vary with increase of circumferential strain at *λ*_*z*_ = 1.5.

Figure 5 plots the stress-strain relations of the mid-wall of coronary arteries at two different axial stretch ratio *λ*_*z*_ = 1.3 and 1.5. With increase of circumferential strain *E*_*θθ*_, compressive radial stress becomes large, but still with small magnitude as compared with other components. The passive circumferential stresses are very low at toe region and then increase rapidly at high strain, while active stresses generated from SMC contraction make the total circumferential stresses comparable even at low strain. The active stresses then gradually decline with increase of strain, but total stresses increase significantly as a result of collagen recruitment. Moreover, SMC contractions increase axial stresses significantly for both *λ*_*z*_ = 1.3 and 1.5, and higher stretch *λ*_*z*_ = 1.5 leads to more increase of axial stress.

We performed a microstructural sensitivity analysis by replacing the continuous spatial distributions of fiber orientation and waviness with a uniform mean orientation angle. We considered two families of elastin with two mean orientation angles (*μ*_*E*1_ and *μ*_*E*2_), and two families of collagen with mean orientation angles (*μ*_*C1*_ and *μ*_*C2*_) with a straightening strain *e*_01_ in adventitia. In media, there are two symmetrical families of SMCs and elastin fibers, while collagen fibers are parallel to elastin fibers and have a different straightening strain *e*_02_. Parameter estimates of fibers and cells are shown in Table 3. The mean-value approach achieved slightly lower R^2^ as compared with that of full distributions.

**Table 3:** Material and geometrical parameter estimates of fibers and SMC determined by a mean-value approach to eliminate continuous distribution of microstructure.

	Parameters	Sample No.					Average ± SD
		1	2	3	4	5	
*Fiber passive*	*k*_*IL*_ (MPa)	0.25	0.0	0.03	0.29	0.22	0.16 ± 0.12
	*k*_*E*_ (MPa)	0.23	0.28	0.21	0.21	0.28	0.24 ± 0.03
	*k*_*C*_ (MPa)	10.5	16.9	10.7	42.7	36.1	23.4 ± 13.5
	*M*_*C*_	5.66	5.66	5.71	5.31	5.13	5.50 ± 0.23
*Fiber geometrical*	*e*_01_	0.23	0.30	0.29	0.22	0.24	0.26 ± 0.03
	*e*_02_	0.39	0.21	0.38	0.36	0.26	0.32 ± 0.07
	*μ*_*E1*_	0.23	0.31	0.36	0.23	0.20	0.27 ± 0.06
	*μ*_*E2*_	1.70	1.62	1.81	1.63	1.90	1.73 ± 0.11
	*μ*_*C1*_	0.38	0.30	0.33	0.40	0.32	0.36 ± 0.04
	*μ*_*C2*_	1.76	1.85	1.74	1.70	1.77	1.76 ± 0.05
	*μ*_*M*_	0.27	0.21	0.26	0.15	0.19	0.22 ± 0.04
*SMC active*	$\,{\rho }_{1}$ (MPa)	0.48	0.36	0.53	0.27	0.24	0.38 ± 0.11
	*ρ*_2_	1.80	1.57	1.73	1.47	1.27	1.57 ± 0.19
	*λ*_*max*_	1.48	1.22	1.48	1.47	1.48	1.43 ± 0.10
	σ_*max*_ (MPa)	0.08	0.10	0.08	0.08	0.12	0.09 ± 0.02
	*τ*	0.13	0.14	0.24	0.17	0.38	0.21 ± 0.09
	*k*_*SMC*_ (MPa)	0.01	0.00	0.00	0.03	0.01	0.01 ± 0.01
*SMC geometrical*	*μ*_*SMC*_	0.26	0.23	0.27	0.20	0.12	0.22 ± 0.05
*Goodness-of-fit of full data*	*R* ^2^ for P	0.93	0.9	0.86	0.99	0.99	0.93 ± 0.05
	*R* ^2^ for F	0.84	0.63	0.70	0.96	0.85	0.80 ± 0.12
	*R* ^2^ for *r*_*o*_	0.96	0.86	0.79	0.96	0.89	0.89 ± 0.06

**Figure 6:**
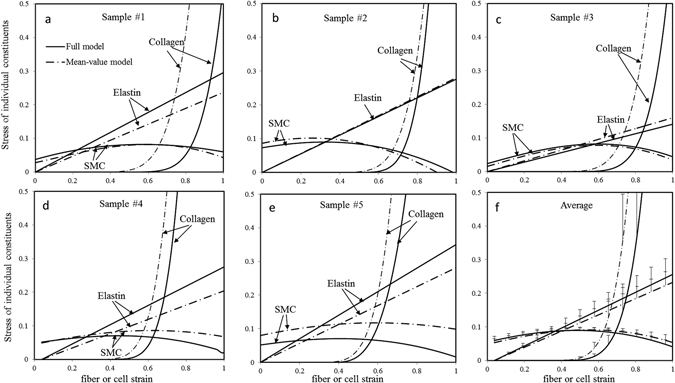
The stress-strain curves of individual fibers and SMC of all samples. Comparisons were made between the full microstructure model and the mean-value approach. Solid lines denote the stresses of fibers and cells predicted by the full model, and dashed lines are predictions of mean-value approach.

Figure 6 shows the stress-strain curves of individual fibers of all samples using material parameters based on full fiber distributions (referred to as ‘full model’) and uniform distributions (‘mean-value model’). In general, elastin fibers take up most of loads at low strain *e* < 0.5, and collagen is then gradually recruited to carry loads at high strain levels. When SMCs contract, they work with elastin to resist loads at low strain, and SMC contraction declines where collagen is then engaged to support the vessel wall. It was found that elastin stiffness determined by the full model is larger than that of mean-value model. For collagen fibers, the stress-strain curve determined by mean-value approach enable earlier fiber recruitment than that of full model, while the active stress-strain curve of SMCs do not show a significant difference between the two models.

## Disscussion

We previously performed comprehensive studies on the biomechanics of coronary arteries, including measurement of geometrical distributions of fibers and SMCs, simultaneously loading-imaging on arterial microstructure, and also integrating these microstructural features into microstructural models of individual media and adventitia layers^1, 5, 6, 11, 18, 21, 22^. Here, a 3D microstructural constitutive model for the coronary arteries with active SMC contractions was proposed to predict the passive nonlinear behaviors, biaxial active responses, and also macro- and micro stress distributions of the vessel wall. To our knowledge, this is the first 3D microstructural active model of coronary arteries with SMC contraction that accounts for all components’ microstructure, including collagen waviness distribution, elastin and collagen orientation distributions in individual media and adventitia, biaxial vasoactivity and symmetrical helical arrangement of media SMCs, as well as isotropic IL elastin distribution in media. It should be noted that although the microstructural distributions employed here were obtained by statistical measurement, rather than animal-specific measurements, this model includes microstructural details which has not been possible previously. Hence, we refer to it as a full constitutive microstructural model. This truly microstructure-based constitutive model has high descriptive and predictive capabilities and is able to provide reliable parameter estimations.

Previous experimental studies^23, 24^ showed that actin filaments largely align in cell direction with a slightly oblique arrangement in isolated SMCs, suggesting that active stress generated by actin-myosin cross-bridge may not only apply in the cell direction (i.e., the major axis of a cell), but also in the transverse direction (minor axes). Moreover, the tensile properties of SMCs freshly isolated from rat aortas measured in both major and minor axes showed that under relaxation, both major and minor axes present very low stiffness of 13.6 ± 10.8 KPa and 1.8 ± 3.0 KPa, respectively, which can be neglected as compared to those of elastin and collagen fibers (with a magnitude of MPa). The stiffness increases significantly in both axes, however, in contracted cells (stimulated by Serotonin) as 92.4 ± 30.1 KPa and 38.3 ± 25.8 KPa, respectively^23, 24^. These observations suggest that contracted cells can present significant stiffness in their minor axes, implying that a one-dimensional active model may not be accurate for contracted cells in a 3D analysis of blood vessels. Moreover, previous phenomenological models suggest symmetric active stress-strain curves due to a symmetric simplification of SMC responses^11, 18, 19^. Some studies dispute this symmetry as they show tension gradually increases prior to a peak and then declines more steeply in a non-symmetric fashion^25–27^. The present work thus proposed a tri-axial asymmetric constitutive law of SMCs in the microstructural model to account for active responses of a single cell under contraction, leading to a better description and predictions of vessel macro- and micro-scopic responses.

For coronary arteries, the majority of collagen fibers in adventitia orient towards the longitudinal direction and the other fibers align nearly in the circumferential direction, following two normal distributions, which is different from previous assumption that fibers are symmetrically disposed with respect to the circumferential direction of the vessel (with a preferred orientation)^3, 28, 29^. The outer radius of passive arteries rapidly increased at low pressure and began to plateau at higher pressures as a result of circumferentially-oriented collagen fibers engaged to withstand loads with increase of pressure (Figs 1 and 3). The axial force was found to increase significantly from *λ*_*z*_ = 1.3 to 1.5 as most longitudinal-oriented fibers were recruited to provoke a rapid increase of axial force at *λ*_*z*_ = 1.5, which was beyond straightening strains of most collagen fibers. The other geometrical parameters, including collagen straightening strain distribution, elastin and SMCs orientations in adventitia and media, respectively, and *IL* elastin distribution (Fig. 2), were also engaged to achieve a full microstructure-based model of coronary arteries. Moreover, geometrical parameters were refined by imposing restrictions to reflect microstructural variation among samples, which provided better predictions of vessel responses (Fig. 3).

The outer radii of coronary arteries are significantly reduced and axial forces increase when SMCs were activated by K^+^ PSS solution, presenting a biaxial active response as showed in Fig. 3a and b. The biaxial vasoactivity of arteries is related to both the helical distributions of SMCs (Fig. 2c) and biaxial SMC vasoconstriction in coronary media. The later was evaluated by the ratio of SMC active axial to circumferential stresses with a mean of *τ* = 0.23 ± 0.08, showing a significant axial active responses. The magnitude of radial stress is much smaller than other components as a result of fiber negligible compressive properties (Fig. 4). The negative stress indicate that tissue is under compression in this direction, and luminal side was compressed more than the outer region. SMC contractions increase transmural stress distributions and the passive stress-strain relations (Figs 4 and 5). At low circumferential strain, constricted SMCs and elastin fiber carry most of loads (Fig. 6). With the increase of strain, collagen fibers gradually engage to take up loads, and SMC contractions gradually decline and active stresses decrease, leading to collagen-dominated nonlinear responses (Figs 5 and. 6). Initial axial stretch *λ*_*z*_ = 1.3 or 1.5 result in a comparable active axial stress even at small circumferential strain (Fig. 5c and f), giving an estimated SMC optimal stretch as *λ*_*max*_ = 1.34 ± 0.12.

The fiber material parameters (*k*_*E*_, *k*_*C*_, *M*_*C*_), which were estimated by the present full model and based on full distension-extension experimental data, show high consistency with that previously estimated by adventitia-specific microstructural model and experimental data^1^, implying that the models deep-rooted in arterial microstructure can provide a reliable estimate of material parameters of individual fibers and cells. The estimated elastin stiffness and collagen parameters are within the reported values of fiber moduli^1, 30–32^, with elastin Young’s modulus between 100 kPa to 1 MPa and collagen tangent modulus with a magnitude of about 10 MPa. The sensitivity analysis of microstructure indicated that the mean-value approach provided a lower elastin stiffness and earlier collagen recruitment. The former was induced by uniformly distributed elastin aligning close to the two major loading directions (i.e., circumferential and axial directions) leading to ‘softer’ elastin fibers, while the later present ‘stiffer’ collagen fiber as a result of uniformly distributed collagen with a unique straightening strain *e*_01_. There were no collagen fibers engaged to withstand loads before fiber strain reached *e*_01_, such that the collagen straightening strain was underestimated to match the overall mechanical response of the vessel wall in the mean-value model. The stress-strain curves of SMCs do not show a significant difference between two models (as shown in Fig. 6) because of that orientation distributions of SMCs are symmetrical along the circumferential direction and disperse relatively narrowly as compared to fibers. The comparison between full model and mean-value approach showed that the accuracy of material parameter estimate; i.e., stress predictions on individual fibers and cells, are highly dependent on the actual distributions of microstructure.

When the focus is on macroscopic behaviors rather than stresses of individual cells and fibers, the mean-value approach model can predict nonlinear responses and stress distribution of the vessel wall, which is the sum of stresses of every constituent, with much higher computational efficiency. Moreover, the mean-value approach model offers reliable predictions for tissues with uniformly distributed fibers^33^. Some other microstructure models, such as ‘four fiber-family’ model^34–37^, can also provide good macroscopic predictions. Assuming four fiber families aligning in the vessel wall, this model showed good agreements between experimental measurements and model predictions as four families almost disperse the entire orientation space of collagen fibers. The models that lack realistic microstructure basis, however, are not able to provide reliable material parameters or to predict micro-stresses on individual fibers and cells. The significance of realistic microstructure features has been appreciated by many recent studies, for which models were proposed based on histology measurements or observations^1, 11, 33, 38–40^. The present study proposes the first 3D microstructure-based active model of coronary arteries accounting for individual fiber and cell distributions in media and adventitia. The microstructural distributions were refined based on previously quantitatively statistic measurement to enable a reliable estimates of material parameters of individual fibers and cells. Conversely, the model also is also capable of predicting microstructural distributions when provided with well-known material properties of the constituents. In brief, the mean value approach, with lower computational expense, provides good predictions of macroscopic mechanical behaviors of blood vessel but is not able to predict reliable material parameters of individual constituents, while a full microstructural model can achieve both goals but requires more computational cost given the greater microstructural detail.

Some limitations of this study should be noted. First, we assume a linear distribution for radial response of contracted SMCs based on the assumption of vessel incompressibility. This is reasonable given the relatively thin wall of the artery (e.g., diameter of artery is an order of magnitude larger than the thickness). Experimental studies on individual SMCs are needed to clarify the radial behaviors of active SMCs. Moreover, the proposed active model is a phenomenological model, of which only *λ*_*max*_ and ${\sigma }_{max}$ are physically meaningful, and a simplification that axial active stress was related to circumferential stress by parameter *τ* has been made. A mechano-chemical 3D constitutive model should be developed to describe SMC contractions in future study.

## Conclusions

The proposed 3D microstructural active model includes microstructural distributions and material properties of adventitial and medial fibers and cells, and thus provides an accurate prediction of passive nonlinear responses, biaxial vasoactivity and transmural stress distributions of the coronary artery arteries. Moreover, with realistic microstructure basis, it enables reliable material parameter estimations of individual elastin, collagen, and SMCs and thus ensures prediction of microscopic stress on fibers and cells. The microstructural model leads to a better understanding of biomechanics of coronary arteries, and can be extended to elucidate the mechanism of vascular disease initiation and progression.

## Materials and Methods

### Sample Preparation

Porcine hearts (n = 5) were obtained at a local slaughterhouse and transported to the laboratory in 4 °C physiological saline solution (PSS) immediately after the animals were sacrificed. The left anterior descending (LAD) arteries were dissected carefully form the hearts and the loose tissues were carefully removed. The LAD segments were then cut ~2 cm in length and kept in 4 °C PSS.

### Distension-Extension Mechanical Test

The specimens were used for pressure distension-axial extension mechanical testing similar to previous studies^41^. A specimen was cannulated on both ends and connected in a testing bath containing PSS at room temperature and aerated with 95% O_2_-5% CO_2_^1, 18^. The internal pressure, axial force and outer diameters can be simultaneously measured by a data acquisition system (MP100, Biopac Systems, Gotleta, CA). The bath temperature was gradually increased to 37 °C in 20 minutes, and the segment was stretched to *in vivo* length (axial stretch ratio *λ*_*z *_≈ 1.3) and pressurized under 20 mmHg for 45 minutes to allow the vessel to equilibrate. The vessel was then preconditioned several times to obtain reproducible mechanical data^42^. The intravascular pressure was increased to 60 mmHg, and the 37 °C PSS was replaced by 80 mM K^+^ PSS at the same temperature, and the vessel was equilibrated for 10 min to attain maximum SMC contraction. The intravascular pressure was gradually increased from 0 to 160 mmHg in increments of 20 mmHg, and two different axial stretch ratios of *λ*_*z*_ = 1.3 and 1.5 were tested. The full pressure-radius and pressure-axial force relations (including both active and passive responses) were measured. After the active test, high K^+^ PSS was removed and the vessel was washed by PSS three times. The vessel was relaxed by Ca^2−^ free PSS for 20 minutes, and a similar testing protocol was repeated and the corresponding passive data was determined. Immediately after measurements, the segment was taken out, and a 2-mm-long ring was cut. The ring was then cut open radially to release the residual stresses and strains i.e., zero-stress state (ZSS) of a specimen, of which the outer and inner radii and opening angles were measured^1, 18, 41^.

### 3D Microstructure-Based Model of Active Coronary Artery

#### Kinematics

**Figure 7:**
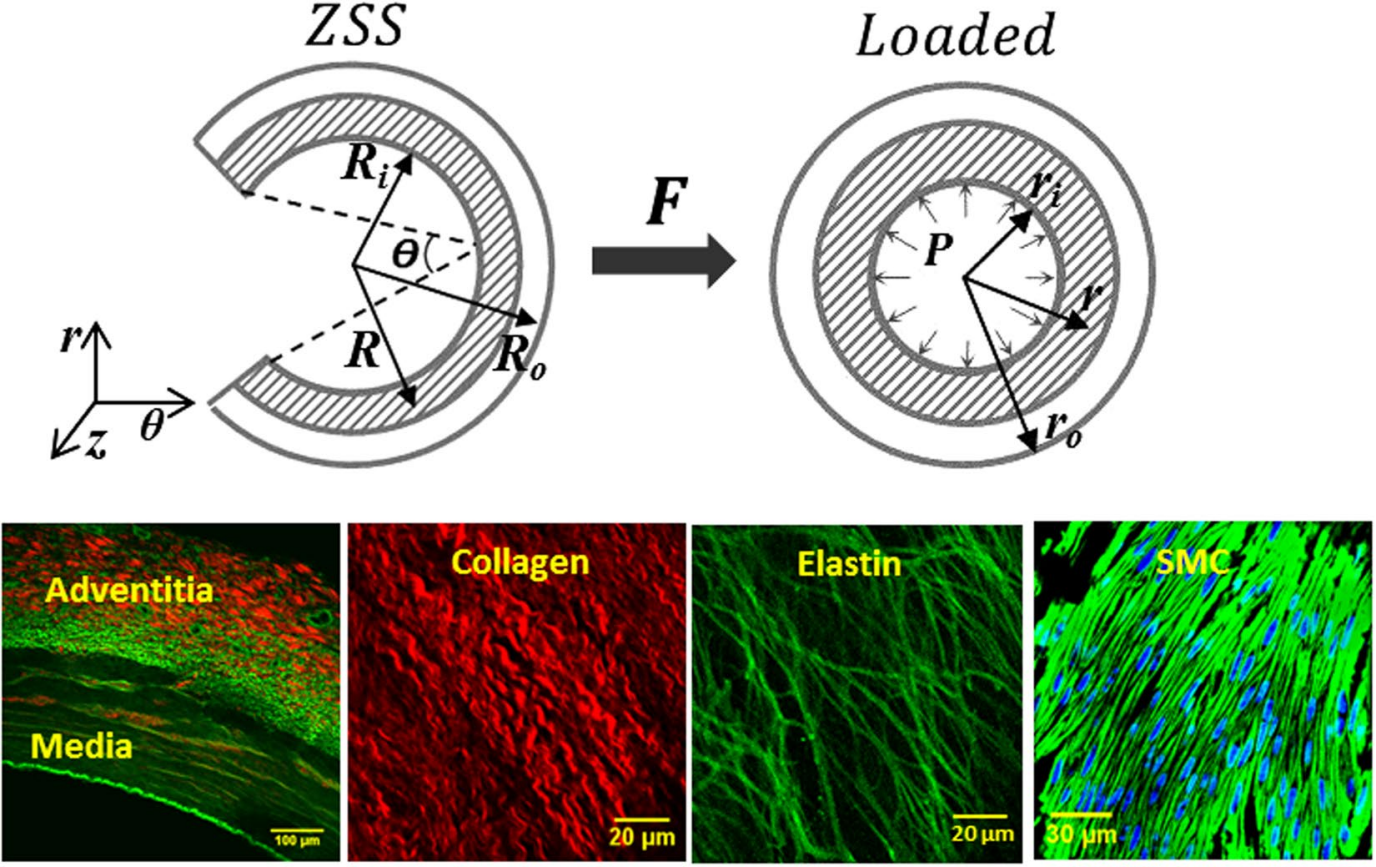
Top: A schematic diagram of vessel configurations and geometrical parameters. The left panel is the coordinate system of vessels with three principal directions: circumferential direction ***θ***, radial direction **r** and axial direction **z;** ZSS is zero-stress state of vessel, and loaded state means vessel under pressure **P**. Deformation gradient tensor **F** describes tissue deformation from ZSS to loaded configuration. Parameters $({R}_{o},{R}_{i}$) are the outer and inner radii of stress-free vessel and *R* is radius to a point measured at ZSS, while ***θ*** is opening angle of vessel at ZSS. Parameters $({r}_{o},{r}_{i}$) are the outer and inner radii of loaded vessel and *r* is radius to the point measured at the loaded state. Bottom: Images of coronary artery and microstructure. Left: multiphoton microscopic (MPM) image of arterial cross-section shows coronary artery layered-structure (Red: collagen; Green: elastin); Middle: MPM images of longitudinal-circumferential sections of collagen and elastin in adventitia, respectively; Right: Confocal images of SMCs in media (Green: F-actin; Blue: cellular nucleus). These images were reproduced from refs. 2, 5 and 11.

Some general kinematic assumptions are considered for a 3D vessel mechanical model^1, 3^: (1) Vessel wall is incompressible; (2) Deformations are axis-symmetric and independent of axial position; (3) Transverse sections remain planar; and (4) There is a unique un-deformed reference configuration (i.e., ZSS). We model the vessel as a cylindrical tube using a cylindrical coordinate system, of which three principal directions are circumferential direction $\overrightarrow{{\boldsymbol{\theta }}}$, radial direction $\overrightarrow{{\boldsymbol{r}}}$ and axial direction $\overrightarrow{{\boldsymbol{z}}}$. The corresponding stretches (*λ*_*θ*_, *λ*_*r*_ and *λ*_*z*_) are given as bellow:

1 $${\lambda }_{\theta }=(\frac{\pi }{\pi -{\Theta }})\,\frac{r}{R},\,{\lambda }_{r}=\frac{\partial r}{\partial R},\,{\lambda }_{z}=\frac{l}{L}$$

where *Θ* is opening angle and *R* is radius to a point measured at ZZS configuration of a vessel, and $r$ is the radius to the same point in the current configuration; i.e., loaded vessel, as showed at top row of Fig. 7. *L* is the axial length of the segment at ZSS and *l* is the loaded axial length. According to material incompressibility, $J={\lambda }_{\theta }{\lambda }_{r}{\lambda }_{z}=1$, for the mapping between ZSS and loaded state, loaded radius *r* was determined as a function of unloaded *R*:

2 $$r(R)=\sqrt{{r}_{o}^{2}-({R}_{o}^{2}-{R}^{2})\frac{\pi -{\Theta }}{{\lambda }_{z}\pi }}$$

where *r*_*o*_ is the outer radius in the loaded state while *R*_*o*_ is that at ZSS. Analogously, *r*_*i*_ and *R*_*i*_ are the inner radii in the loaded state and ZSS, respectively.

The radial component of the force equilibrium equation imposed on the loaded configuration is given by:

3 $$\frac{\partial {\sigma }_{rr}}{\partial r}+\frac{{\sigma }_{rr}-{\sigma }_{\theta \theta }}{r}=0$$

where ${\sigma }_{rr},\,\,{\sigma }_{\theta \theta }$ are the radial and circumferential components of Cauchy stress, respectively. According to boundary conditions ${{\sigma }_{rr}|}_{{r}_{i}}=-{p}_{i},\,{{\sigma }_{rr}|}_{{r}_{o}}=0$, the luminal pressure ${p}_{i}\,\,$can be written as:

4 $${p}_{i}={\int }_{{r}_{i}}^{{r}_{o}}({\sigma }_{\theta \theta }-{\sigma }_{rr})\frac{1}{r}dr\,$$

The axial tension can be determined by the integration of the axial components of Cauchy stress, ${\sigma }_{zz}$, over the cross-sectional area:

5 $$N=2\pi {\int }_{{r}_{i}}^{{r}_{o}}{\sigma }_{zz}rdr=F+{p}_{i}\pi \,{r}_{i}^{2}$$

where axial force is determined as $F=\pi {\int }_{{r}_{i}}^{{r}_{o}}(2{\sigma }_{zz}-{\sigma }_{z\theta }-{\sigma }_{rr})rdr$, and ${p}_{i}\pi \,{r}_{i}^{2}$ accounts for the internal pressure in the closed tube during the test. The circumferential and axial equilibrium equations under distension-extension loading yield all shear stress components $\,{\sigma }_{r\theta }={\sigma }_{z\theta }=\,{\sigma }_{zr}=0$. Therefore, three components of Cauchy stress were provided by the 3D model, of which the radial component ${\sigma }_{rr}\,$was not able to be predicted by a 2D model^11, 18^.

The coronary artery is considered as an incompressible hyper-elastic solid and characterized by a strain energy function (SEF) $W({\bf{E}})$ as a function of the Green-Lagrange strain tensor ${\bf{E}}=\frac{1}{2}({{\bf{F}}}^{{\rm{T}}}\cdot {\bf{F}}-{\bf{I}})$. The Cauchy stress tensor **σ** is given by^43^:

6 $${\boldsymbol{\sigma }}={\bf{F}}\frac{\partial W}{\partial {\bf{E}}}{{\bf{F}}}^{{\rm{T}}}-p{\bf{I}}={\bf{F}}\cdot {\bf{S}}\cdot {{\bf{F}}}^{{\rm{T}}}-p{\bf{I}}$$

where **F** is the deformation gradient tensor and **S** is the second Piola-Kirchhoff stress tensor. **I** is the second order identity tensor, scalar *p* is hydrostatic pressure, which acts as a Lagrange multiplier and must be determined from equilibrium and boundary conditions.

### Microstructure Features of Active Coronary Arteries

Many microstructure-based constitutive models have been developed with various microstructural assumptions or simplifications^3, 28, 29^. A true microstructural model should be rooted in actual ultra-structural measurements of vessel wall constituents. Accordingly, we have quantitatively measured the elastin, collagen and SMC geometrical distributions in the wall of coronary arteries in several previous studies. We experimentally confirmed that the ground substance plays a negligible role in mechanical support of coronary adventitia under tension and hence, can be considered as a fluid-like matrix^1^. We employed simultaneous mechanical loading-imaging to quantify *in situ* deformation of individual collagen and elastin fibers on unstained fresh LAD adventitia to confirm that undulated collagen fibers are recruited to bear loads only after they become straightened under high pressures^5, 6^. Moreover, we quantitatively measured geometrical distributions of fibers and cells in individual adventitia and media layers^5, 6, 11^.

Based on these previous microstructural studies (as showed Fig. 7), the following axioms have been integrated to synthesize a realistic microstructural constitutive model: (1) A fluid-like matrix is employed and suggests the tissue undergoes affine deformations. Therefore, the SEF of the vessel wall can be represented by the volume-weighted summation of individual SEFs of every constituent. (2) All fibers are only resistant to tensile load and have negligible compressive and bending rigidities. Elastin fibers take up most of load at low pressures, while collagen fibers gradually become straightened and are recruited with an increase of pressure. (3) In adventitia, many collagen fibers oriented toward the axial direction and the others aligned nearly in the circumferential direction following a mixture of two normal distributions. The orientation of elastin fibers also follows two normal distributions where the minor orientation of elastin was approximately orthometric to the major orientation. (4) The straightening strain of adventitia collagen was found to follow a beta distribution with a mean and standard deviation of (0.35, 0.051). (5) In media, most SMCs arrange in θ-z (circumferential-axial) plane and slightly aligned off circumferential direction of blood vessels with symmetrical polar angles, and the axial active response of blood vessels is associated with SMC biaxial contraction. (6) The majority of media fiber bundles are also planar and their orientations are consistent with but not exactly identical to that of SMCs^15^. Finally, there exist isotropic inter-lamellar (IL) elastin networks in media, which disperse over orientation space including the radial direction^3, 15^.

Based on the above axioms, the total SEF of vessel wall is a sum of volume-weighted SEFs of fibers and SMCs:

7 $$W({\boldsymbol{E}})={f}_{IL}{W}_{IL}+{f}_{E}{W}_{E}+{f}_{C}{W}_{C}+{f}_{SMC}{W}_{SMC}$$

where ${f}_{E}$, *f*_*IL*_, *f*_*C*_ and ${f}_{SMC}$ are volume fractions of elastin, collagen and SMC, respectively.

### Passive SEF of Coronary Artery

Although the SMCs are the predominate contributors of active behavior, they provide negligible contributions to the passive properties of blood vessels^23, 44, 45^. The passive SEF is thus only determined by SEFs of elastin and collagen fibers:

8 $${W}_{P}({\boldsymbol{E}})={f}_{IL}{W}_{IL}+{f}_{E}{W}_{E}+{f}_{C}{W}_{C}$$

The SEF of a fiber family is a function of fiber orientation $\theta$ as below ($i=IL,\,E,C$):

9 $${W}_{i}={\int }_{0}^{\pi }{ {\mathcal R} }_{i}(\theta ){w}_{i}(e)d\theta $$

where ${ {\mathcal R} }_{i}(\theta )$ is the orientation distribution density function of fiber *i*, and ${w}_{i}(e)$ is the SEF of a single fiber. The uniaxial fiber strain $e(\theta )$ is determined by the local strain tensor **E** and the reference fiber direction **N** as: $e({\bf{E}},{\bf{N}})={\bf{E}}:{\bf{N}}\otimes {\bf{N}}$. It should be noted that ${ {\mathcal R} }_{i}(\theta )$ satisfies the normalization criterion ${\int }_{0}^{\pi }{ {\mathcal R} }_{i}(\theta )d\theta =1\,$.

In adventitia, orientations of elastin and collagen fibers follow a mixture of two normal distribution of fiber^6^ (*i* = *E*, *C*):

10 $${W}_{Ai}=\sum _{j}^{2}{\omega }_{ij}{\int }_{0}^{\pi }{ {\mathcal R} }_{ij}(\theta ){w}_{i}(e)d\theta $$

where ${ {\mathcal R} }_{ij}(\theta )=\frac{1}{{{\rm{K}}}_{ij}}\frac{1}{{\sigma }_{ij}\surd 2\pi }\,Exp[-\frac{{(\theta -{\mu }_{ij})}^{2}}{2{\sigma }_{ij}^{2}}],\,(i=E,C;j=1,2)$ is a truncated normal distribution density function with ${\mu }_{ij}$ and ${\sigma }_{ij}$ as the mean and standard deviation, respectively. ${{\rm{K}}}_{ij}$ is the total weight of the truncated normal distribution ${{\rm{K}}}_{ij}={\Phi }(\frac{\pi -{\mu }_{ij}}{{\sigma }_{ij}})-{\Phi }(\frac{-{\mu }_{ij}}{{\sigma }_{ij}})\,$(*Φ* is the cumulative distribution function of a normal distribution)^1^, and ${\omega }_{ij}$ is the weight of each normal distribution $({\sum }_{j}^{2}{\omega }_{ij}=1)$.

In media, elastin and collagen fibers aligned off of circumferential direction of blood vessels, following two symmetric normal distributions that are analogous to SMCs. We assume both fibers have the same distributions ${ {\mathcal R} }_{E}(\theta )={ {\mathcal R} }_{C}(\theta )={ {\mathcal R} }_{M}(\theta )$, which is a normal distribution density function with mean and standard deviation (${\mu }_{M}$, ${\sigma }_{M}$). Moreover, media *IL* elastin is an isotropic network, the overall SEF of the passive media can be thus written as (*i* = *E*, *C*):

11 $${W}_{Mi}=\{\frac{1}{2}{\int }_{0}^{\frac{\pi }{2}}{ {\mathcal R} }_{i}(\theta ){w}_{i}(e)d\theta +\frac{1}{2}{\int }_{-\frac{\pi }{2}}^{0}{ {\mathcal R} }_{i}(-\theta ){w}_{i}(e)d\theta \}+\frac{1}{\pi }{\int }_{-\frac{\pi }{2}}^{\frac{\pi }{2}}{w}_{IL}(e)d\theta $$

According to microscopic responses of individual elastin fibers under mechanical loads^5, 6^, the elastic properties was assumed to be linear, thus the SEF of elastin is given by:

12 $${w}_{E}=\frac{1}{2}\,{k}_{E}{e}^{2}$$

where fiber strain *e* is larger than zero (fiber is only resistant to tensile load), and *k*_*E*_ is stiffness parameter of elastin fiber. *IL* elastin has a similar function but with a different stiffness *k*_IL_:

13 $${w}_{IL}=\frac{1}{2}\,{k}_{IL}{e}^{2}$$

Because of the wavy nature of collagen fibers, the SEF was considered to account for the nonlinear elastic behavior^3^:

14 $${w}_{C}=\frac{1}{1+{M}_{C}}\,{k}_{C}{(e-{e}_{0})}^{1+{M}_{C}}$$

where fiber strain *e* is larger than collagen straightening strain *e*_0_, beyond which the collagen can withstand tension (which also denotes fiber waviness). *k*_*C*_ and *M*_*C*_ are parameters characterizing the nonlinear stress-strain response of collagen. It was found that collagen straightening strain ${e}_{0}\,$follows a Beta distribution in LAD adventitia^6^:

15 $$D({e}_{0})=\frac{1}{B({\alpha }_{1},{\alpha }_{2})}\frac{{({e}_{0}-a)}^{{\alpha }_{1}-1}{(b-{e}_{0})}^{{\alpha }_{2}-1}}{{(b-a)}^{{\alpha }_{1}+{\alpha }_{2}-1}}\,$$

where $B({\alpha }_{1},{\alpha }_{2})$ is a Beta function, and *a* and *b* the lower and upper bounds of the straightening strain *e*_0_. A uniform distribution of straightening strain was assumed for media collagen since collagen bundles are thinner and have a lower volume fraction as compared to adventitia. The material properties of elastin and collagen fibers were assumed to remain constant throughout the vessel wall.

### Active stresses of Coronary Artery with SMC contraction

Since the force of SMC contraction is generated by active energy consuming processes, a stored elastic energy (i.e., SEF), which is a function of strain state, is not appropriate. An empirical length-tension relationship is typically employed in this case. Most 2D active models suggest uniaxial length-tension relationships in the circumferential direction as motivated by the circumferential arrangement of SMCs^12–15^. In present work, a 3D model is proposed to account for tri-axial responses of a single cell to better predict the overall behavior of blood vessels. A phenomenological stress-strain law was employed in the cell direction (i.e., major axis of a cell) as^26^:

16 $${\sigma }_{SMC}=\,A\,[\frac{\,{\rho }_{1}}{2}({\lambda }_{max}^{{\rho }_{2}}-{\lambda }_{SMC}^{{\rho }_{2}}-2){({\lambda }_{SMC}-{\lambda }_{max})}^{2}+{\sigma }_{max}]$$

where *A* is the level of activation (0 is passive state and 1 is fully active, A=1 for the present study), ${\lambda }_{SMC}=\sqrt{{\boldsymbol{n}}\cdot ({{\bf{F}}}^{{\boldsymbol{T}}}\cdot {\bf{F}})\cdot {\boldsymbol{n}}}$ is the longitudinal stretch of a SMC (i.e., cell stretch), ***n*** and ***n***′ are the longitudinal and transversal vector, respectively. ${\lambda }_{max}$ denotes an optimal stretch ratio at which a SMC generated maximum stress ${\sigma }_{max}$. *ρ*_1_ and *ρ*_2_ determine the curvature and the skewness of the curve, respectively. When ${\rho }_{2} > 0$, the absolute slope of lower stretch region (${\lambda }_{SMC}\le {\lambda }_{max}$) is smaller than that of high stretch region (${\lambda }_{SMC} > {\lambda }_{max}$), while ${\rho }_{2} < 0$ leads an inverse behavior; and *ρ*_2_ = 0 provides a symmetric curve as employed in previous models.

An analogous relation was used to account for SMC transverse stress in one of minor axes (there are two minor axes of a single cell: transverse and radial axes) as:

17 $$\,{\sigma }_{SMC}^{^{\prime} }=\tau \,A[\frac{{\rho }_{1}}{2}({\lambda }_{max}^{{\rho }_{2}}-{\lambda }_{SMC}^{^{\prime} {\rho }_{2}}-2){({\lambda }_{SMC}^{^{\prime} }-{\lambda }_{max})}^{2}+{\sigma }_{max}]$$

of which ${\lambda }_{SMC}^{^{\prime} }=\sqrt{{\boldsymbol{n}}{\boldsymbol{^{\prime} }}\cdot ({{\bf{F}}}^{{\boldsymbol{T}}}\cdot {\bf{F}})\cdot {\boldsymbol{n}}{\boldsymbol{^{\prime} }}}$ is SMC stretch in transverse direction, and $\tau$ is a dimensionless parameter, that determines the contractile properties in this direction, which can be regarded as the ratio of active axial stress to circumferential stress.

In the radial direction of a cell, a similar stress-strain law should be considered. SMCs, however, are largely compressed in the radial direction and stretched in other two directions under current distention-extension loading conditions. Moreover, Eq. (16) is a strongly non-linear and non-monotonic function, of which a large span in stretch variable may lead to divergence of the solutions. A linear law stress-strain was thus considered as a simplification of the stiffness of active SMCs in the radial direction as:

18 $$\,{\sigma }_{SMC}^{^{\prime\prime} }={k}_{SMC}{\lambda }_{SMC}^{^{\prime\prime} }$$

of which ${\lambda }_{SMC}^{^{\prime\prime} }$ is the radial stretch of SMCs which is equivalent to the radial stretch ratio of the vessel wall as SMCs arrange in $\theta -z$ plane, and *k*_*SMC*_ is the stiffness in radial direction during SMC contraction.

The second Piola-Kirchhoff stress of each constituent was thus derived as:

19 $${{\boldsymbol{S}}}_{i}=\frac{{\rm{\partial }}W({\boldsymbol{E}})}{{\rm{\partial }}{\bf{E}}}=\sum _{i}^{IL,\,E,\,C,\,SMC}{\omega }_{ij}{\int }_{0}^{\pi }\,{{\mathcal{R}}}_{ij}(\theta )\frac{{\rm{\partial }}{w}_{i}}{{\rm{\partial }}e}\frac{{\rm{\partial }}e}{{\rm{\partial }}{\bf{E}}}d\theta $$

and the total stress is the volume-weighted sum as given by:

20 $${\boldsymbol{S}}={f}_{IL}{{\boldsymbol{S}}}_{IL}+{f}_{E}{{\boldsymbol{S}}}_{E}+{f}_{C}{{\boldsymbol{S}}}_{C}+{f}_{SMC}{{\boldsymbol{S}}}_{SMC}$$

of which ${f}_{IL}{{\boldsymbol{S}}}_{IL}+{f}_{E}{{\boldsymbol{S}}}_{E}+{f}_{C}{{\boldsymbol{S}}}_{C}$ presents passive second Piola-Kirchhoff stress of vessel wall. ${f}_{SMC}{{\boldsymbol{S}}}_{SMC}$ is the active stress generated by SMC contraction, of which the components can be written as:

21 $${S}_{SMC\_ij}={\sigma }_{SMC}\frac{\partial {\lambda }_{SMC}}{\partial {{\rm{E}}}_{ij}}+\,{\sigma }_{SMC}^{^{\prime} }\frac{\partial {\lambda }_{SMC}^{^{\prime} }}{\partial {{\rm{E}}}_{ij}}+\,{\sigma }_{SMC}^{^{\prime\prime} }\frac{\partial {\lambda }_{SMC}^{^{\prime\prime} }}{\partial {{\rm{E}}}_{ij}}$$

The Cauchy stress components (i.e., Eq. (6)) of the vessel will then be obtained by substituting the constitutive laws for individual fibers and cells (Eqs. 13–18, 21) into Eq. (20).

The full microstructural model has 15 geometrical parameters and requires 9 material parameters of individual fibers and cells, given the volume fractions of fibers and cells that measured in our previous studies^2, 5, 46^. We have quantitatively measured orientation and undulation distribution parameters of fibers and cells and obtained statistical distributions based on two groups (one for adventitia and another for media) of coronary artery specimens^5, 6, 11^. These statistical measured parameters were directly integrated into the model to predict mechanical responses of additional porcine LAD arteries (n = 5) performed in this study. The 9 material parameters were determined by optimization with appropriate boundary condition (Eqs. 4 and 5). Moreover, we refined microstructural geometrical parameters for each sample by imposing restrictions to ensure fiber orientation and waviness still follow statistic distributions measured in previous studies^2, 5, 6, 11^ and obtained a better agreement between model predictions and experimental data.

### Parameter Estimation

Parameters were optimized by least squares fit to the experimental data by minimizing an objective function based on the sum of squared residuals (SSE) between model predictions and experimental data. In generally, passive and active parameters were determined separately. The passive material parameters of fibers were first determined by pressure-radius and pressure-force relations, and subsequently integrated into the active model to determine active parameters of SMCs. These passive parameters, however, were determined in a limited loading range; i.e., range of only passive loading conditions. For instance, the outer diameters of coronary arteries decreased significantly due to SMCs contractions at very low pressure as shown in Fig. 1a and b. The passive parameters, determined under axial stretch ratio *λ*_*z*_ = 1.3 and circumferential stretch ratio *λ*_*θ*_ from 0.9 to 1.8 (corresponding pressure varying from 20 mmHg to 160 mmHg), were integrated into the active model to determined active parameters under *λ*_*z*_ = 1.3 and lower ${\lambda }_{\theta }$ from 0.6 to 1.7. Thus, the active parameters were under- or over-estimated somewhat as passive parameters were not validated in the range of ${\lambda }_{\theta }$ from 0.6 to 0.9, which was an important region of vessel active response (i.e., pressure varying from 20 to 60 mmHg as shown in Fig. 1a and b). Therefore, an objective function simultaneously accounting for both passive and active behaviors was defined as follows:

22 $$SSE=\frac{1}{nm}\sum _{i,j}^{n,m}[{(\frac{{r}_{oij}^{P}-{\widehat{r}}_{oij}^{P}}{{\sigma }_{{\hat{r}}_{o}}^{P}})}^{2}+{(\frac{{F}_{ij}^{P}-{\hat{F}}_{ij}^{P}}{{\sigma }_{\hat{F}}^{P}})}^{2}+{(\frac{{r}_{oij}^{F}-{\widehat{r}}_{oij}^{F}}{{\sigma }_{{\hat{r}}_{o}}^{F}})}^{2}+{(\frac{{F}_{ij}^{F}-{\hat{F}}_{ij}^{F}}{{\sigma }_{\hat{F}}^{F}})}^{2}]$$

where *i* and *j* denote distension and axial loads at which the corresponding outer radius (${r}_{oij}^{P}$, ${r}_{oij}^{F}$) and axial force (${F}_{ij}^{P}$, ${F}_{ij}^{F}$) were measured. *P* denotes passive responses and *F* denotes full responses (including both passive and active) of coronary arteries, *n* is the number of different pressures and *m* is the number of different axial stretch ratios used. ${\sigma }_{\hat{\cdot }}$ is the standard deviation of experimental measurement, and ${\widehat{{r}_{o}}}_{ij}\,$, ${\hat{F}}_{ij}$ are corresponding model predicted outer radius and axial force. The objective function with more restrictions (i.e., typically separated into 2 objective functions) leads to a better identification of material parameters; especially, for the active parameters. A Genetic Algorithm method was employed to search optimal parameter sets using Fortran language executed in Linux.
